# Regression of Stage IV Pancreatic Cancer to Curative Surgery and Introduction of a Novel Ex-Vivo Chemosensitivity Assay

**DOI:** 10.7759/cureus.423

**Published:** 2015-12-21

**Authors:** Mayrim V Rios Perez, Bingbing Dai, Eugene J Koay, Robert A Wolff, Jason B Fleming

**Affiliations:** 1 Department of Surgical Oncology, The University of Texas MD Anderson Cancer Center; 2 General Surgery, University of Puerto Rico; 3 Department of Radiation Oncology, The University of Texas MD Anderson Cancer Center; 4 Department of GI Medical Oncology, The University of Texas MD Anderson Cancer Center

**Keywords:** metastatic pancreatic cancer, stage iv pancreatic cancer, curative surgery, pancreaticoduodenectomy, ex-vivo chemosensitivity assay, folfirinox

## Abstract

Although data suggests little hope for survival when patients present with metastatic pancreatic cancer, recent advances in systemic therapy offer the possibility for dramatic tumor responses like those observed in other disease sites. Here, we present the case of a 50-year-old woman who presented with adenocarcinoma of the pancreas with two liver metastases and a CA 19-9 level of 1,659 U/mL. The patient received FOLFIRINOX (leucovorin, 5-fluorouracil, irinotecan, and oxaliplatin) with a dramatic reduction in CA 19-9 level to 23.9 U/mL, and complete regression of both liver metastases. The patient then received capecitabine with the maintenance of a normal CA19-9 over the next 12 months. With no evidence of distant disease, concurrent systemic and local therapy with capecitabine-based chemoradiation (CapeXRT) was performed followed by observation for eight months with normal CA 19-9 readings. A mild increase in CA 19-9 (143 U/mL) prompted a restaging demonstrating an active primary tumor but no distant disease. Therefore, a pancreaticoduodenectomy (PD or Whipple) was performed rendering this patient free of detectable cancer.

Our team has developed an ex-vivo chemosensitivity assay in which the tumor tissue from an individual patient can be rapidly examined for sensitivity to available systemic therapy treatment strategies. We tested this patient’s tumor for its sensitivity to gemcitabine (Gem) versus a combination of 5-fluorouracil, irinotecan, and oxaliplatin (FIRINOX). Remarkably, our assay confirmed a profound sensitivity of this patient’s tumor to the agents she had received.

## Introduction

Pancreatic adenocarcinoma remains an unsolved health care dilemma, in large part because most patients present with distant metastasis and an anticipated survival of three to 11 months [1-6]. Even though a common scenario, this patient population is relatively understudied with regard to therapy. For example, many clinical trials investigating new agents include patients with locally advanced disease (localized tumors that are unresectable) along with patients with distant metastasis. These are biologically distinct patient groups with unique response rates to therapy [7-8].

The current standard of care chemotherapeutic regimens for metastatic pancreatic cancer include FOLFIRINOX and gemcitabine, plus nab-paclitaxel (gemcitabine/nab-paclitaxel) based upon positive Phase III trials where the tumor response rate was 33% and 23%, while the progression-free survival was 6.4 and 5.5 months, respectively. The equipoise of these two strategies presents a decision dilemma for oncologists as there is currently no effective method for choosing one approach over the other. The patient in the case presented below, who consented for publication, as well as tissue collection under IRB approved protocols, LAB00-396 and PA15-0176, demonstrates that exceptional responses can be achieved in patients presenting with metastatic pancreatic cancer when effective systemic therapy is combined with well-executed local treatment modalities.

## Case presentation

**Figure 1:**
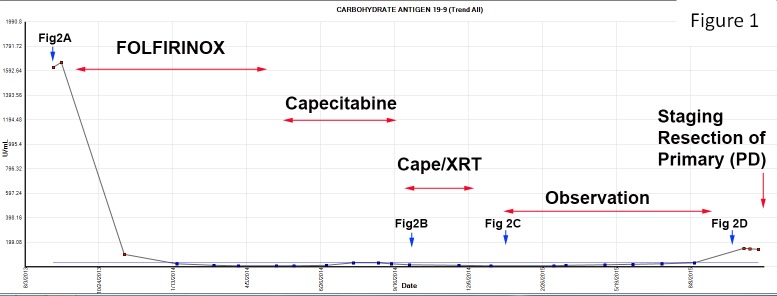
Serum carbohydrate antigen 19-9 (CA 19-9) trend showing treatment effect over time. CapeXRT: capecitabine-based chemoradiation.

**Figure 2:**
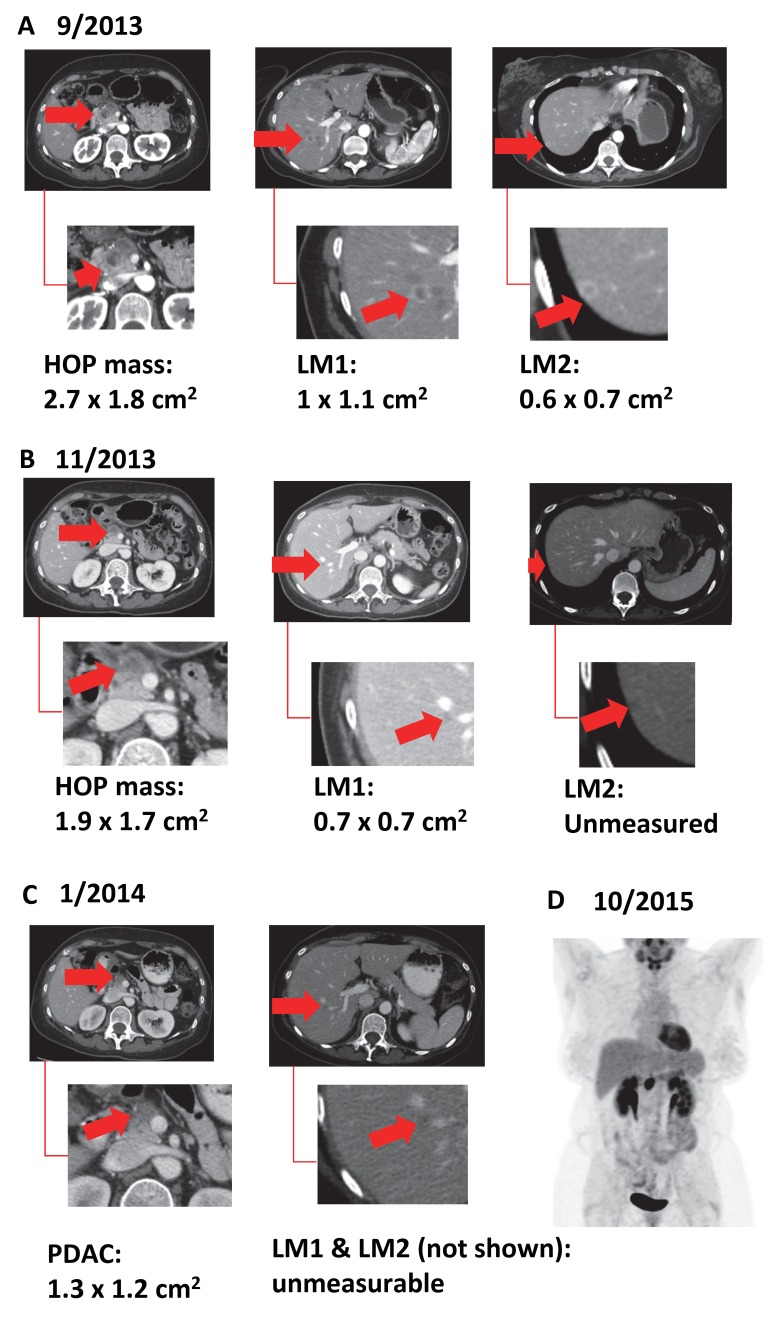
Series of CT scans showing treatment effect over lesions from diagnosis (A) followed by FOLFIRINOX, Capecitabine (B), and CapeXRT (C) treatment. FDG-PET scan showing single pancreatic lesion (D) HOP: head of the pancreas, LM1: liver metastasis #1, and LM2: liver metastasis #2, PDAC: pancreatic adenocarcinoma, CapeXRT: capecitabine-based chemoradiation.

A 50-year-old woman presented with a complaint of abdominal pain for several months that was not relieved by a laparoscopic cholecystectomy. This prompted a CT scan, which identified a lesion on the head of the pancreas (HOP), and endoscopic ultrasound-directed biopsy confirmed this to be primary pancreatic ductal adenocarcinoma (PDAC), along with an elevated serum CA 19-9 level of 1,659 U/mL (Figure 1). A staging pancreas protocol CT scan was performed at our institution the following month, which identified a 2.7 x 1.8 cm^2 ^mass in the pancreas and two liver masses (LM1 and LM2) consistent with metastatic tumors (Figure 2A). The markedly elevated CA 19-9 observed at presentation was also consistent with metastatic disease. After discussion at a multidisciplinary conference, the patient received six cycles of FOLFIRINOX over three months with removal of oxaliplatin the last month. During this time, the patient’s pain dissipated and the CA 19-9 level normalized from 1,659 to 23.9 U/mL (Figure 1); additionally, staging CT scans demonstrated regression of both hepatic lesions (Figure 2B). After this response, the patient received maintenance capecitabine over four months, during which time complete resolution of the liver lesions was observed (Figure 2C), along with maintenance of a resectable primary tumor and a persistently normal CA 19-9. After multidisciplinary review, the patient received concurrent local and systemic therapies with capecitabine-based chemoradiation (CapeXRT): 30 Gray delivered to the primary tumor in 10 fractions combined with one gram of oral capecitabine twice a day on a Monday through Friday schedule (days of radiation only), managed by the radiation oncologist and the GI medical oncologist, respectively. The patient was then observed over the next eight months until an elevation in serum CA 19-9 was identified (143 U/mL) (Figure 1). This prompted a CT and FDG-PET scan (Figure 2D) that identified an enlarging and FDG-avid primary tumor with no metastasis. Staging laparoscopy was performed with biopsy of identified lesions in the liver and peritoneum demonstrating fibrosis with no cancer cells present. After careful counseling with the patient and discussion with the team, a PD was performed as a potentially curative operation. The patient recovered without complication and pathologic examination of the specimen identified a 1.8 x 1.8 cm^2^ invasive, moderately differentiated adenocarcinoma without metastatic lymph nodes (0/24). In summary, the application of effective systemic and multimodal local therapy reduced this patient from a Stage IV initially to Stage IIB (T3N0M0) at the time of resection. This downstaging has generated 28 months of survival to date.

## Discussion

To our knowledge, this is the first report of pancreatic cancer metastatic to the liver downstaged by FOLFIRINOX followed by local therapy with chemoradiation and an R0 pancreatic resection. This case demonstrates how an exceptional response can be achieved when an available systemic treatment regimen is matched to the patient with a tumor sensitive to the component agents. Our laboratory is researching new methods that could provide the patient-specific data necessary for the clinician to make a timely and accurate decision when choosing gemcitabine/nab-paclitaxel versus FOLFIRINOX for a patient with Stage IV pancreatic cancer.

**Figure 3:**
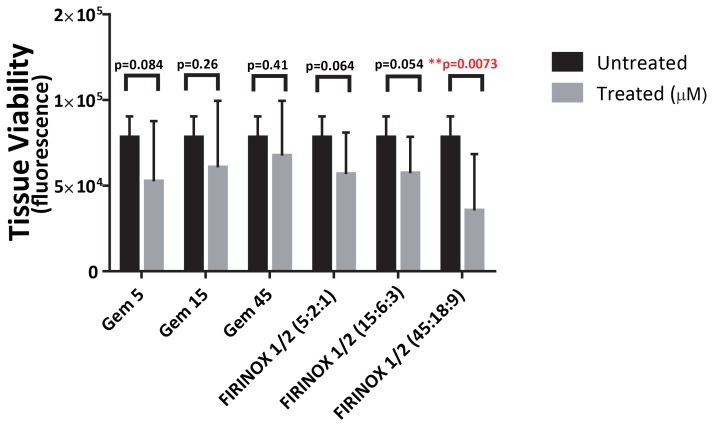
Ex-vivo chemosensitivity assay: gemcitabine versus FIRINOX. PrestoBlue assay as per manufacturer's instructions was used to determine viability (expressed as fluorescence) of the tissue slices placed on a 96-well plate after they were obtained from fresh patient tumor cores. Tissue slices were treated with three doses of gemcitabine (Gem) and the combination: 5-fluorouracil, irinotecan and oxaliplatin (FIRINOX), respectively. Data was obtained by using FLUOstar Omega microplate reader 48 hours after treatment. Untreated slices (0.5% DMSO) were used as a negative control. Analysis was done using GraphPad Prism 6 by comparing treated versus untreated groups using multiple t-tests with p<0.05 defined as statistically significant.

This experimental method involves harvesting a core of tumor tissue that can be precision-sliced into discs deployed on a 96-well culture plate and tested for sensitivity to either treatment regimen. Importantly, the results of this could be available for interpretation and reporting to the clinician within 48-hours of tissue harvest. Results from this assay in this patient’s primary tumor are displayed in Figure 3. This data demonstrates that this patient’s tumor sample was very sensitive to the combination: 5-fluorouracil, irinotecan, and oxaliplatin (FIRINOX) when compared to gemcitabine, a finding which closely mirrors this patient’s remarkable response to FOLFIRINOX. We are developing this assay as part of a clinical trial that will prospectively test its ability to identify the most effective treatment approach for individual patients presenting with untreated Stage IV pancreatic cancer.

This case also provides a glimpse into the future of how advanced pancreatic cancer could be treated if effective systemic therapies are available, as is currently the case for many patients with metastatic colorectal cancer (CRC). Survival was less than 12 months prior to the development of effective systemic therapies, but now patients with metastatic CRC, even hepatic metastases, often survive five years or longer as clinicians learn to match combinations of cytotoxic-targeted therapy to patient tumor types susceptible to those agents. The effective systemic therapy is then combined with well-performed surgical resection of residual tumor sites [9-10]. Together, the reported data from metastatic CRC and this case underscore a critical point: achieving survival benefit after effective systemic therapy requires the addition of safe and oncologically sound ablation or removal of all residual viable cancer sites. This was performed in our case when the FDG-PET, CT imaging, and laparoscopic biopsies identified that the remaining site of viable pancreatic cancer resided in the primary tumor. Once identified, surgical removal necessitated performance of a PD. This procedure carries a significant risk of morbidity and mortality, which could obliterate any improvement in survival and patient quality of life obtained by the response to chemotherapy. Fortunately, the procedure-related risks can be mitigated when the procedure is performed at high-volume centers, underscoring the necessity of high-quality care by all members of the multidisciplinary team.

## Conclusions

This case report exemplifies how state-of-the-art multidisciplinary therapy for a patient with metastatic pancreatic adenocarcinoma can result in downstaging to surgical resection. Additionally, this case provides a hopeful glimpse into a future state in which more precise application of systemic therapy will allow many patients to reap the same survival benefit.
